# Optimizing Outcomes in Pedicle and Free Flap Reconstruction in Patients With Sickle Cell Trait

**Published:** 2018-01-08

**Authors:** David Buziashvili, Richard S. Zeri, Tom Reisler

**Affiliations:** ^a^New Jersey Medical School, Rutgers University, Newark; ^b^Division of Plastic and Reconstructive Surgery, Department of Surgery, The Brody School of Medicine, East Carolina University, Greenville, NC

**Keywords:** sickle cell trait, free flap, pedicel flap, thrombosis, exchange transfusion

## DESCRIPTION

A 46-year-old African American man presented with grade IIIB Gustilo-Anderson open left distal tibia/fibula fracture, which was sustained after a 10-ft fall into a ditch. The patient underwent a successful anterolateral thigh (ALT) perforator free flap reconstruction, anastomosed to the posterior tibial vessels.

## QUESTIONS

1. What is sickle cell and can patients with sickle cell trait (SCT) undergo erythrocyte sickling and under what conditions?
2. What are the potential systemic complications displayed in persons with SCT and why?
3. What are the potential associated flap complications in patients with SCT undergoing microsurgical or pediceled flap reconstruction?
4. What measures can be taken to reduce flap complications in patients with SCT?

## Discussion

Sickle cell disease (SCD) is a relatively common disorder of hemoglobin (Hb) formation inherited through autosomal recessive means with a prevalence of 80,000 Americans. It is a single nucleotide substitution of GTG to GAG at the sixth codon of the β-globin gene, replacing the amino acid glutamate to valine.^1^ The morphology of the red blood cell (RBC) is altered during periods of deoxygenation from a normal biconcave (HbA) to a sickle shape (HbS). HbS presents with a rigid and more viscous RBC. These irregularly shaped cells can cluster together and obstruct blood vessels. This can trigger a series of events that lead to tissue damage and eventual inflammatory response, causing the vaso-occlusions and painful crises that are hallmark of SCD.^1^ It was previously thought that patients with SCT, having only one diseased allele of HbS and milder to no symptoms, were regarded as healthy. However, with enough systemic stressors such as severe tissue hypoxia, acidosis, dehydration, and hypothermia, erythrocyte sickling can occur and cause similar problems as in SCD.^2^ Even under normal conditions, SCT RBCs are inherently stiffer and more viscous than normal RBCs.^3^

Physiologically, individuals with SCT have higher levels of coagulative factors such as D-dimers, thrombin-antithrombin complexes, and monocyte levels, which mediate endothelial cell damage and may promote thrombogenesis. Studies have shown that SCT patients are significantly more vulnerable to venous thromboses and further venous thromboembolism, as they can have up to 45% of their total Hb as HbS.^4^^,^^5^ Patients with SCT are 4 times more likely to develop pulmonary embolism when compared with the healthy population. In addition, SCT patients have double the risk of ischemic stroke incidents.^6^^,^^7^

Patients with SCT are at risk of flap failure due to a combination of flap microcirculatory sludging precipitated by systemic or local stressors and hypercoagulability.^4^

Several measures can be taken to reduce flap-related complications due to sickling within the flap microcirculation in SCT patients. Pedicel flaps are thought to be safe when the patient's HbS is maintained between 30% and 40% .^5^ Preoperative prophylactic measures such as an exchange transfusion to reduce HbS levels remain advocated but alone is ineffective in preventing flap thrombosis.^4^ In addition, an indirect approach through hydroxyurea treatment combined with erythropoietin will increase levels of fetal Hb and reduce HbS formation.^1^ Data show that ischemia time during flap elevation may cause flap failure. By keeping ischemia time as short as possible during free tissue transfer (flap off-time), or alternatively by choosing a well-vascularized pedicel flap, one can achieve a reduction in flap complications. It has been speculated that free flap tissue hypothermia induced by cooling, a method used for flap preservation during free flap transfer, may precipitate erythrocyte sickling within the flap microvasculature and may be the leading cause of intraflap thrombosis.^2^ Flap survival may be potentially improved by minimizing or avoiding direct local tissue hypothermia or systemic hypothermia. Adequate systematic oxygen saturation and hydration throughout surgery and postoperatively are essential. Limiting vasoconstrictors will reduce flap necrosis as well. Introducing hyperbaric oxygen therapy at the time of initial signs of ischemia may be beneficial as well.^2^^,^^5^

In conclusion, surgical and anesthesia guidelines exist for patients with SCD to prevent sickle cell crisis; however, no guidelines are established for SCT patients as they are regarded as healthy.^2^ Clearly, SCT patients are also at risk of flap necrosis complications and appropriate precautions must be taken.^5^^,^^8^

## Figures and Tables

**Figure 1:**
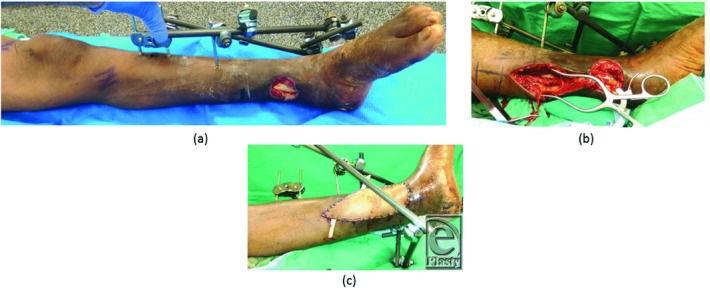
(a) Photograph demonstrating a grade IIIB Gustilo-Anderson open left distal tibia/fibula fracture; (b) photograph with the display of the posterior tibial recipient vessels; and (c) photograph with the anterolateral thigh perforator free flap inset.

**Figure 2:**
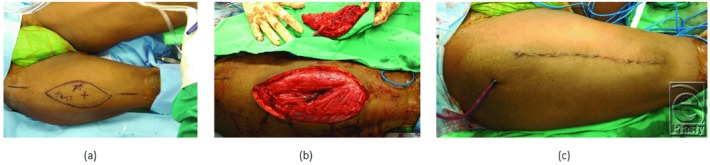
(a) Preoperative marking of the right anterolateral thigh perforator free flap, 17 × 7 cm in size; (b) harvesting of the free flap; and (c) primary closure of the donor site.

## References

[1] Conran N, Franco-Penteado CF, Costa FF (2009). Newer aspects of the pathophysiology of sickle cell disease vaso-occlusion.. Hemoglobin.

[2] Spear SL, Carter ME (2003). Low M, Duck I, Schwartz J. Sickle cell trait: a risk factor for flap necrosis. Plast Reconstr Surg.

[3] Zheng Y, Cachia MA, Ge J, Xu Z, Wang C, Sun Y (2015). Mechanical differences of sickle cell trait (SCT) and normal red blood cells. Lab Chip.

[4] Han KD, DeFazio MV, Lakhiani C, Evans KK (2015). Free tissue transfer in patients with sickle cell trait: not just a trait. Plast Reconstr Surg.

[5] Platt AJ, Robertson A, Batchelor AG (2000). Successful free flap transfer and salvage in sickle cell trait. Br J Plast Surg.

[6] Austin H, Key NS, Benson JM, et al (2007). Sickle cell trait and the risk of venous thromboembolism among blacks. Blood.

[7] Caughey MC, Loehr LR, Key NS, et al (2014). Sickle cell trait and incident ischemic stroke in the Atherosclerosis Risk in Communities Study. Stroke.

[8] Davison SP, Kessler CM, Al-Attar A (2009). Microvascular free flap failure caused by unrecognized hypercoagulability. Plast Reconstr Surg.

